# Classical Microbiological Diagnostics of Bacteremia: Are the Negative Results Really Negative? What is the Laboratory Result Telling Us About the “Gold Standard”?

**DOI:** 10.3390/microorganisms8030346

**Published:** 2020-02-29

**Authors:** Tomasz Źródłowski, Joanna Sobońska, Dominika Salamon, Isabel M. McFarlane, Mirosław Ziętkiewicz, Tomasz Gosiewski

**Affiliations:** 1 Thoracic Anesthesia and Respiratory Intensive Care Unit, John Paul II Hospital, 31- 202 Kraków, Poland; t.zrodlowski@szpitaljp2.krakow.pl; 2 Department of Internal Medicine, St. John’s Episcopal Hospital, Far Rockaway, NY 11691, USA; 3 Department of Molecular Medical Microbiology, Chair of Microbiology, Faculty of Medicine, Jagiellonian University Medical College, 31-121 Krakow, Poland; 4 Department of Medicine, SUNY Downstate Medical Center, Brooklyn, NY 11203, USA; 5 Department of Anesthesiology and Intensive Care, Faculty of Medicine, Jagiellonian University Medical College, 31-501 Krakow, Poland

**Keywords:** sepsis, bacteremia, blood culture, Gram staining, Fluorescence in-situ Hybridization (FISH)

## Abstract

Standard blood cultures require at least 24–120 h to be reported as preliminary positive. The objective of this study was to compare the reliability of Gram staining and fluorescent in-situ hybridization (FISH) for detecting bacteria in otherwise negative blood culture bottles. Ninety-six sets were taken from patients with a diagnosis of sepsis. Six incomplete blood culture sets and eight blood cultures sets demonstrating positive growth were excluded. We performed Gram stain and FISH on 82 sets taken from post-operative septic patients: 82 negative aerobic blood cultures, 82 anaerobic blood cultures, and 82 blood samples, as well as 57 blood samples taken from healthy volunteers. From the eighty-two blood sets analyzed from the septic patients, Gram stain visualized bacteria in 62.2% of blood samples, 35.4% of the negative aerobic bottles, and in 31.7% of the negative anaerobic bottles. Utilizing FISH, we detected bacteria in 75.6%, 56.1%, and 64.6% respectively. Among the blood samples from healthy volunteers, FISH detected bacteria in 64.9%, while Gram stain detected bacteria in only 38.6%. The time needed to obtain the study results using Gram stain was 1 h, for FISH 4 h, and for the culture method, considering the duration of growth, 5 days. Gram stain and FISH allow quick detection of bacteria in the blood taken directly from a patient. Finding phagocytosed bacteria, which were also detected among healthy individuals, confirms the hypothesis that blood microbiome exists.

## 1. Introduction

Sepsis is “a life-threatening organ dysfunction caused by a dysregulated host response to infection” as defined by the Society of Critical Care Medicine and European Society of Intensive Care Medicine Task Force (SCCM/ESICM Sepsis Definitions Task Force). The preliminary diagnosis is usually based on the clinical picture and is often made before any laboratory results become available [1], while the diagnostic and prognostic assessments are based on sepsis-related organ failure assessment (SOFA) or the quick SOFA (qSOFA) scores [2]. The SOFA score includes: PaO2/FiO2, the need of mechanical ventilation, Glasgow Coma Scale, mean arterial pressure, platelet count, bilirubin and creatinine level.

The global incidence of sepsis is difficult to ascertain due to lack of or insufficient reporting, but it ranges from 73.6/100,000 in the United States in 1979 to 1180/100,000 in Northern Australia from 2007 to 2008 [3,4]. In 2016, the global incidence of sepsis was estimated at 31 million cases per year (437 per 100,000) and its mortality, around 5 million/year. Being that sepsis is the main cause of mortality worldwide [5], the prompt identification of the source of infection and determination of the etiologic agent are important for optimization of therapy, including the selection of an effective, targeted antibiotic therapy, which can contribute to improved prognosis in patients with sepsis.

The detection of bacteria in the blood can be difficult due to a variety of causes ranging from the relatively small number of organisms at a given time, their periodic seeding into the bloodstream, and the antibiotic therapy applied which can remain in the culture media reducing the chances of recovering microorganisms in the cultures. The current gold standard in microbiological diagnostics involves culturing blood samples in automated systems such as BACTEC (Becton Dickinson). The flaws of the blood culture method include the time needed to detect growth, which can take up to several days, and its low sensitivity, leading to detection of microbial growth in the range 15–61% of cases with 30–50% on average [6,7].

Attempts have been made to enhance the effectiveness of detection of etiologic agents using serological and molecular methods. Serological methods identify microbial components, such as lipopolysaccharide (LPS) of Gram-negative bacteria or mannan and galactomannan of fungi [8,9,10], while molecular methods detect the microbial genetic material in a shorter time and with higher precision than the traditional culture method [11,12]. Molecular methods do not require microbial growth in a culture medium and do not depend on previous antibiotic therapy [13,14,15].

Serological and molecular diagnostics are, however, expensive and require special expertise. Therefore, screening methods could be very beneficial as they would facilitate the detection of bacteria (bacteremia) and fungi (candidemia) in the blood or, allow a higher efficacy of microorganism detection especially in standard blood culture that failed to show growth [16,17]. Among the screening methods, Gram staining and FISH (fluorescence in-situ hybridization) are useful as they allow the visualization of microbial cells in the direct smear [13,18,19,20].

Due to the poor recovery rate of the standard blood culture method, the objective of this study was to assess if the gold standard method may omit the presence of bacteria in some of the tested blood samples. We aimed to ascertain not only if detection of bacteremia is feasible utilizing Gram stain and FISH on reportedly negative blood cultures and blood samples of healthy volunteers but also to compare these two screening methods.

## 2. Materials and Methods

### 2.1. Patients

The study was conducted at the John Paul II Specialist Hospital in Cracow, Poland, which admits over 20,000 patients/year, and performs about 2000 open heart procedures and 1100 thoracic procedures annually.

Blood samples (2 mL) and standard blood culture were taken from patients admitted to the Intensive Care Unit (ICU) (Table 1). The study was conducted from August 1, 2017 to December 31, 2018. The patients who took part in the study had undergone thoracic surgery subsequently developing post-operative pulmonary complications for which intravenous antibiotics had been initiated. During the course of the ICU stay, the patients demonstrated new signs and symptoms of sepsis (SOFA ≥ 2); laboratory tests were ordered including blood cultures and other basic labs. In total, 96 septic patients with a SOFA score ≥ 2 points were enrolled in the study. SIRS score and inflammatory markers (CRP, procalcitonin) were monitored but were not a part of the inclusion criteria of the study. Exclusion criteria were SOFA score < 2, incomplete blood sets, and growth of bacteria in blood cultures. The 57 healthy volunteers were recruited by advertisements posted in the hospital’s Bulletin Board. Inclusion criteria was the absence of inflammation markers (CRP, leukocytosis).

Ethics consideration: All subjects gave their informed consent for inclusion before they participated in the study. The study was conducted in accordance with the Declaration of Helsinki, and the protocol was approved by the Jagiellonian University Bioethical Committee (No. 1072.6120.131.2017) on 28 September 2017.

### 2.2. Samples

In total, 96 blood set were taken from septic patients with a SOFA score ≥ 2 points [2]: 2–3 blood sets were taken from different anatomical sites for standard microbiological diagnostics. A single set consisted of blood taken for aerobic and anaerobic bacterial cultures and a separate 2 mL blood sample. Under strict sterile conditions, blood cultures (aerobic and anaerobic bottles-FAN Plus media (bioMérieux) with adsorbent polymeric beads (APB) to neutralize antimicrobials) were drawn (10 mL each) from the patients with sepsis and inoculated into the BacT/ALERT^®^3D system (bioMérieux, Marcy-l’Étoile, France) blood culture media. During the same phlebotomy, a 2-mL blood sample was also collected from the septic patients into VACUETTE^®^ TUBE K3E (Becton Dickinson, Eysins, Vaud. Switzerland).

Fifty-seven 2-mL blood samples were drawn into VACUETTE^®^ TUBE K3E (Becton Dickinson, Eysins, Vaud. Switzerland) from healthy volunteers under sterile conditions.

The blood culture bottles were sent promptly to the Microbiology laboratory and placed in the BacT/ALERT^®^3D system (bioMérieux, Marcy-l’Étoile, France). The 2-mL blood samples from the septic patients and the healthy volunteers were stored at −72 °C until further analysis. After 5 days of incubation, the negative blood culture media were also frozen at −72 °C and maintained until enough samples had been collected prior to working in batches.

The next step was defrosting of the 2-mL blood samples and the blood culture media samples using a warm bath at 37 °C. After defrosting, the samples were ready to undergo the FISH method and Gram staining according to the procedures described below.

### 2.3. Blood Purification, Pellets Separation, and Direct Smear Preparation

Utilizing the methodology described by Gosiewski et al. (2017), erythrocytes and hemoglobin (creating a strong background) were removed from the blood samples and culture media using ammonium chloride solution. The next phase consisted of sample concentration by centrifugation. The obtained pellet of leukocytes was then used to prepare the microscope slides for Gram staining and FISH [21,22].

### 2.4. Gram Staining

The standard Gram stain technique was carried out and the smears were examined using the CX21 light microscope (Olympus, Olympus Corporation, PA, USA 18034-0610).

### 2.5. Fluorescence in-situ Hybridization (FISH).

The blood samples were subjected to the FISH methodology previously described by our group, which allows the visualization of bacterial DNA or localized bacterial RNAs within cells [11,13]. The FISH technique can be summarized as follows: The leukocyte pellet sample was fixed utilizing 4% paraformaldehyde, followed by a 96% solution of methanol, air drying, application of the probe, denaturation by heating at approximately 50 °C, removing the unbound probe and applying 4′,6- diamidino-2-phenylindole (DAPI) (Sigma, Poznan, Poland) which is a fluorescent stain that binds strongly to adenine–thymine rich regions in the DNA. To detect bacteria in the samples, the following probes labelled with fluorophores were employed (Genomed, Warsaw, Poland): to detect all bacterial species-probe EUB338: (FITC-5′-GCT GCC TCC CGT AGG AGT-3′-FITC) [23], for the genus *Staphylococcus*-probe STA: (CY3- 5′–TCC TCC ATA TCT CTG CGC 3′) [24], and for the *Enterobacteriaceae*-probe ENT183: (CY3- 5′-CTC TTT GGT CTT GCG ACG-3′) [25]. Leukocytes were stained using the DAPI dye (Sigma). The probes encompassed over 95% of bacterial taxa present in the blood of patients with sepsis. FISH smears were examined using a BX51 fluorescence microscope (Olympus) equipped with a 100x immersion objective, a UV lamp (Olympus, Olympus Corporation, PA, Corporation, USA 18034-0610) and an F-View camera (Olympus, PA, USA, 18034-0610). Image analysis was carried out with the use of AnalySYS (Soft Imaging, Warsaw, Poland) computer software.

### 2.6. Statistical Analysis

The results were subjected to statistical analysis using parametric and nonparametric tests using IBM SPSS Statistics Software, ver. 25 (IBM Corp., NY,10504-1722 USA). Categorical variables were examined using the chi-square test or Fisher’s exact test. Continuous variables were analyzed using the t-test. Statistical hypotheses were verified at the significance level of *p* < 0.05.

## 3. Results

The patients who took part in the study met the SOFA criteria of sepsis after a thoracic surgery. The mean SOFA score ranged from 6.6 in patients with positive blood cultures to 7.9 in patients with negative blood cultures.

From the 96 sets taken from patients with a diagnosis of sepsis, six incomplete blood culture sets and eight blood culture sets demonstrating positive growth were excluded. The following microorganisms were found in blood samples excluded from the study: *Staphylococcus aureus, S. epidermidis* (two patients)*, Klebsiella pneumoniae, Streptococcus mitis, S. haemolyticus, Lactobacillus* spp., *Candida albicans*.

Therefore, 82 blood sets from the sepsis group (2-mL blood samples plus the aerobic and anaerobic bottles) and 57 blood samples from the control group were analyzed using both Gram stain and FISH.

**Figure 1:**
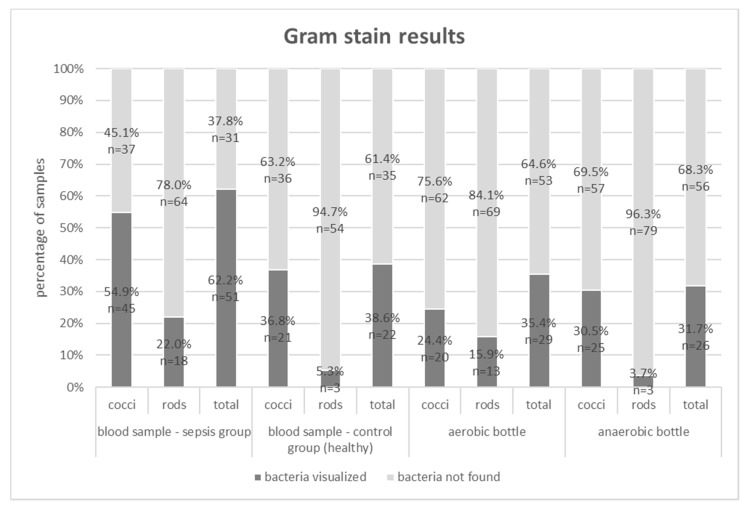
Gram stain results for blood samples, aerobic and anaerobic bottles in the group of patients with sepsis and for blood samples in the control group; (*n*)—absolute values of visualized bacteria.

**Figure 2:**
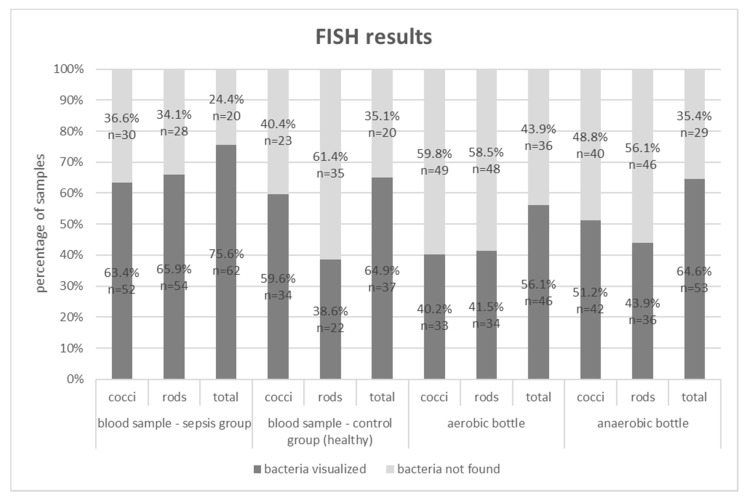
FISH results for blood samples, aerobic and anaerobic bottles in the group of patients with sepsis and for blood samples in the control group; (*n*)—absolute values of visualized bacteria.

Below, in Figure 1 and Figure 2, the results of the study (Gram stain and the FISH method) are presented for the septic patients and the healthy volunteer groups.

“Total” for Gram stain and the FISH methods indicates that bacteria, including cocci, rods, and others were visualized. In the analyzed samples, Gram-negative and Gram-positive bacteria were often isolated simultaneously (e.g Gram staining of blood samples in sepsis group visualized cocci in 54.9% and rods in 22%). Hence, the sum of results is higher than the total positive results (62.2%).

**Figure 3:**
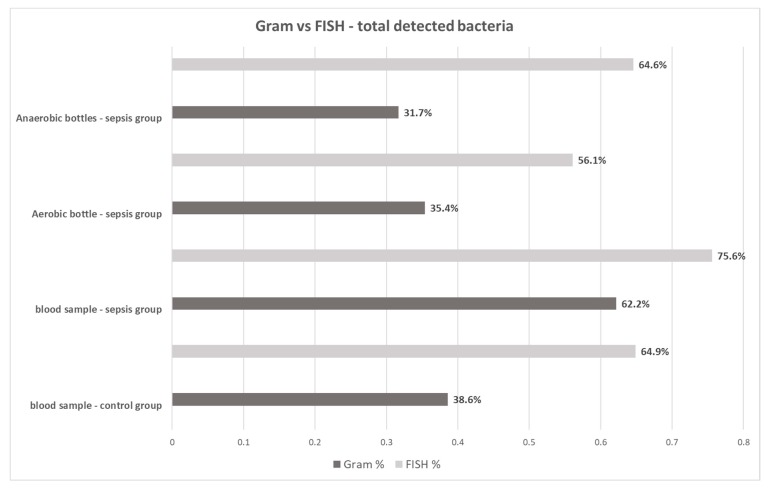
Total Gram stain and FISH results for blood samples, aerobic and anaerobic bottles in the group of patients with sepsis and for blood samples in the control group.

The Gram stain technique was able to detect bacteria in 62.2% (51/82) of 2-mL blood samples from the sepsis group, 35.4% (29/82) of aerobic bottles, 31.7% (26/82) of anaerobic bottles, and in 38.6% (22/57) of 2-mL blood samples from the control group. The FISH methodology detected bacteremia in 75.6% (62/82) of 2-mL blood samples, 56.1% (46/82) of aerobic bottles, 64.6% (53/82) of anaerobic bottles and in 64.9% (37/57) of 2-mL blood samples from the control group (Figure 3). For each type of sample, FISH gave a higher percentage of positive results than the Gram staining method (Table 2). The differences were statistically significant (*p* < 0.05).

**Figure 4:**
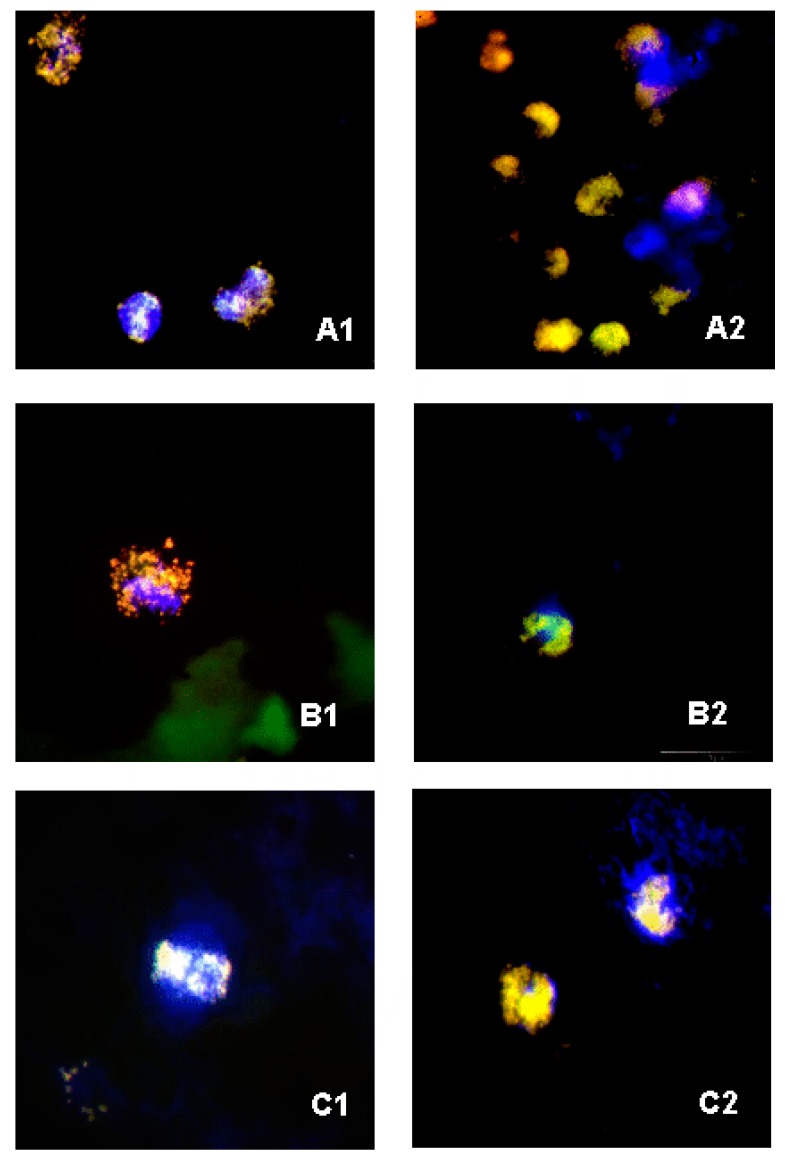
FISH photos from a fluorescent microscope: (**A1**,**A2**) blood sample from septic patients— smear with probes STA and EUB338—visible phagocytosed *Staphylococcus* spp. cells; (**B1**,**B2**) negative samples in blood culture media—smear with probes STA, ENT183 and EUB338—visible phagocytosed *Staphylococcus* spp. and *Enterobacteriaceae* cells; (**C1**,**C2**) blood sample from healthy volunteers—smear with probes STA and EUB338 smear with probes STA, ENT183 and EUB338— visible phagocytosed *Staphylococcus* spp. and *Enterobacteriaceae* cells. 1000x zoom.

On the basis of microscopic image analyses of FISH smears from the blood, negative bottles derived from patients with sepsis and the blood from the control group, it was found that, in all the cases, there were clusters of bacterial cells which had undergone phagocytosis, visible in leukocytes stained blue with the use of DAPI (Figure 4). As for the smears that were Gram stained, phagocytic cells were not visualized (due to the nonspecific dye); however, there were clusters of bacterial cells.

Concordance of results obtained from Gram stain and FISH was also analyzed. The result was deemed concordant if, when applying both research methods, it was possible to visualize bacteria in the same sample or when no bacteria was found in the same sample. In the group with sepsis, the results were concordant in 86.6% for blood samples, 67.1% for anaerobic bottles, and 79.3% for aerobic bottles; in the control group, results were concordant in 73.68% for blood samples. The concordance of Gram stain and FISH was statistically significant (*p* < 0.05) for all variants analyzed in both groups.

Correlation was also studied between the SOFA score (mean 6, range 17), the levels of CRP (mean 194, range 644) and procalcitonin (mean 6, range 102), as well as the frequency of positive or negative results for the smears tested; however, the examined parameters did not show statistically significant dependencies (*p* > 0.05).

The time necessary to complete the testing using Gram stain was 1 h, while for FISH, it was 4 h.

## 4. Discussion

Experts agree that sepsis defines a collection of organ failure symptoms caused by infection [26]. Sepsis is a set of symptoms caused by systemic inflammation due to bacterial or fungal infection. Symptomatic bacteremia does not necessarily mean sepsis, but it is classified as a blood stream infection (BSI) and requires microbiological diagnostic of blood.

Until now, the blood culture has been the gold standard with regard to detecting bacteria in blood. The advantages of this method lie in its undeniable simplicity, low costs, and the possibility to determine the susceptibility/resistance of bacteria to antibiotics. The unquestionable flaws are its sensitivity and the long time needed for bacterial growth. Positive blood cultures in patients with sepsis were found in 15–61% of cases, 30–50% on average [6,7].

A low proportion of positive bacterial cultures, the discovery of endo- and exotoxins and the phenomenon of systemic inflammatory response led researchers to the conclusion that sepsis is not always accompanied by bacteremia [8,9,10], but its appearance is associated with a much higher mortality [27]. The approach to this issue is changing with the modifications to the definition of sepsis and the developments regarding diagnostic methods. The application of molecular biology techniques in diagnostics, among others, new-generation sequencing based on analysis of 16S rDNA, made it possible to discover new phenomena. The most surprising fact was the discovery of genetic material of bacteria in the blood of all subjects, both healthy volunteers and patients with sepsis [12,27,28]. However, in healthy volunteers mostly anaerobic bacteria DNA was found, while in septic patients the predominance of aerobic bacteria DNA was observed. The term “blood microbiome” has even been invented to refer to the taxonomic diversity of the numerous traces of bacteria that are present in the blood [28,29]. Their location and forms can vary. Some are identified following lysis of erythrocytes [30,31] (L-forms), dysfunctional in terms of the cell wall and cell division but showing activity [32]. Some of them are present in the intracellular form in phagocytes and are referred to as neutrophil-associated microbiomes (NAMs), and such a form was identified in our study using the FISH method. Neutrophil dysfunction may lead to excessive release of bacterial material from phagocytes, which will overpower their phagocytosis and change the image of the blood microbiome [32,33,34].

Traditional methods in automated blood culture systems detect only living bacteria, capable of cell division, while the Gram stain and molecular methods can confirm the presence of cell structures or their nucleic acids, without information on the metabolic activity of cells [17]. In our study, we attempted to increase the detectability of bacteria directly in the blood and post-culture fluids using Gram stain and FISH with the application of the methodology that was devised by our team. Eight (8.9%) out of 90 of the collected blood culture sets demonstrated growth. The low percentage of positive cultures compared to global data may have probably been associated with the use of antibiotics prior to blood sample collection for microbiological testing. The clinical characteristics of the group of patients excluded from the study, due to positive blood culture results, is similar to the study group, therefore the condition of the patients could not affect the culture results (Table 1). Patients who took part in the study had undergone thoracic surgery, in which infectious pulmonary complications were predominant. For that reason, the patients had received intravenous antibiotics before the development of septic symptoms, which might have influenced the results of blood cultures, but not the results obtained through Gram stain or FISH [13]. Despite the antibiotic therapy applied and the fact that the amount of blood drawn for testing was modest, both methods allowed the visualization of bacteria in the blood of those patients whose cultures gave negative results (Figure 1, Figure 2 and Figure 3). Moreover, these methods also enabled us to detect bacteria in post-culture fluids, which were considered negative by the automated blood culture system; therefore, a portion of the results may probably be classified as false negatives.

It was very interesting to learn that, in the case of all the samples in which the presence of bacteria was confirmed through the application of FISH, it was possible to visualize large amounts of bacterial cells enclosed in leukocytes stained with DAPI (probably due to phagocytosis), which may explain the lack of bacterial growth (Figure 4). Also, 65% of blood samples drawn from healthy volunteers showed, owing to FISH, the presence of phagocytosed bacteria (Figure 2, Figure 3 and Figure 4). Our observations are confirmed by the study by Li et al. (2018), which showed the presence of the aforementioned specific neutrophil-associated microbiome in healthy individuals and patients with sepsis [29]. It cannot be ruled out that, in the case of sepsis, there are so many bacteria in the blood that phagocytosis is not effective enough, hence, bacterial antigens stimulate the inflammatory process. On the other hand, in a healthy adult, bacteria are quickly phagocytosed and have no opportunity to trigger inflammation, but they can be detected using molecular biology methods. Results of the Gram stain did not demonstrate the presence of phagocytes (given the lack of specific staining), however, they allowed the presence of clusters of bacterial cells to be revealed.

The greatest controversy regarding the application of highly sensitive research methods is the possibility of obtaining false positive results through contamination of the sample, usually with microorganisms that can be found on the skin. Collecting an uncontaminated blood sample is the key stage when carrying out a blood culture, Gram staining, as well as molecular methods. A false positive is defined as growth of bacteria in a blood culture bottle when they are absent in the patient’s bloodstream. The source of the contamination may be the patient’s skin, but also the hands of the person who is taking the sample, the tools utilized, or the immediate surroundings. Microorganisms such as *Corynebacterium*, *Cutibacterium*, coagulase-negative staphylococci, or Bacillus spp. are often present on the skin, but they rarely cause serious infections. Finding them in a single blood culture may be treated as contamination of no clinical significance. One should also pay attention to the fact that coagulase-negative staphylococci may be the cause of catheter-related bloodstream infection (CRBSI) and central line-associated bloodstream infection (CLABSI) or may be the etiologic agent behind bacteremia or sepsis. Bacterial cultures may show growth of bacteria released from the biofilm that covers vascular catheters and from other sources, e.g., bone infections [35,36]. Furthermore, for all samples that showed the presence of bacteria owing to FISH, bacterial cells were visualized enclosed in phagocytes, which proves that they did not come from the outside, hence, they were not a result of contamination (Figure 4).

Detection of bacterial cells in blood samples (especially in healthy volunteers) might be a consequence of transient bacteremia. In the literature, its descriptions appear most often in the context of invasive dental or surgical procedures. While the study group of patients (with clinical symptoms of sepsis) underwent thoracic surgery, the control (healthy) group did not undergo any surgical intervention. It is very difficult to clearly determine the percentage of false positive blood test results because there are no specific determinants of contamination—it is only possible to determine a higher or lower probability which could have been higher in the study group.

There are few studies that confirm the possibility of bacterial detection in negative blood culture fluids, with the use of an additional culture on enriched media or using Gram stain, however, due to the lack of a procedure for concentrating or purifying the samples or the use of FISH, the authors obtained lower percentages of bacterial detection compared to our results [37,38]. In the literature, there are no reports on the detection of bacteria directly in the blood using FISH, apart from the studies by our team, so we cannot compare the obtained results with other studies [13,20]. With the use of FISH, a high percentage of genetic material of Gram-negative bacteria in the blood samples of healthy individuals was detected, which would argue for the translocation of bacteria into the bloodstream and, generally, for the existence of bacterial DNAemia phenomenon, in completely healthy people [39,40,41]. The presence of bacterial ribosomal RNA signals the presence of bacterial cells, hence, also their DNA, since ribonucleic acid degrades very quickly, in contrast to deoxyribonucleic acid. Elevated levels of circulating free DNA (cfDNA) in the blood that come directly from the breakdown of bacterial or phagocytic cells as well as resulting from necrosis or apoptosis have been described in pathological condition (sepsis, inflammation, stroke, cancer), physiological conditions (pregnancy, physical exertion), and following surgical procedures [41,42,43].

The studies are based on the detection of microbial DNA using state-of-the-art molecular methods such as NGS (Next Generation Sequencing) or PCR (Polymerase Chain Reaction), which identify specific fragments of microbial DNA. These techniques, however, do not answer the question of whether the detected DNA was contained in a living or dead cell, or whether it is the result of the breakdown or release of DNA into the bloodstream by phagocytic cells. FISH and Gram staining may detect dead bacterial cells inside phagocytic cells which can explain the detection of bacteria in the negative blood cultures and in the blood taken from healthy volunteers. Non-specific binding of Gram dyes or probes to non-bacterial targets might occur, but it is easily distinguished from the shape of bacterial cells. Owing to the blood sample purification method that was applied by our team, it was possible to obtain a high-quality microscopic image, which allowed us to directly visualize the Gram stained bacterial cells, but also was an opportunity to detect genetic material inside bacterial cells, which confirms our previous observations [20,21,22].

The FISH and Gram staining methods allowed the detection of bacterial cells but did not provide information about whether the cells were alive or dead. It is possible that in the study group, in which clinical signs of infection were present, live bacterial cells were detected, but in the control group, dead bacteria could probably be found. The use of diagnostic methods, which do not determine the status of the microorganism cells, may lead to a clinically false positive result. On the other hand, blood culture (detection of live cells) often fails, despite the presence of clinical signs of blood stream infection. FISH methodology detects in-situ bindings of the nucleic acid probe, which is supposed to be specific to the segment of a bacterial genome, while Gram staining visually detects the morphology of the microscopic image which supports the presence of the bacterial cell. However, without proper negative control, one does not guarantee analytical specificity. Repetitive testing (10 times for example) over the same “seemingly” negative or positive blood samples might give a statistical indication of the analytical specificity of this methodology. Therefore, clinical assessment of the patient’s condition is critical and in this context the analysis of the results of microbiological blood tests is very important.

We also attempted to correlate the SOFA score, procalcitonin levels, CRP, or the leukocyte count with the frequency of positive results of Gram stain or FISH, but the results did not show statistical significance. Lack of correlation of the clinical picture and the levels of inflammatory parameters with the frequency of the detection of bacterial material can be explained by the presence of microorganisms in both healthy and septic individuals, which is also confirmed by other researchers [12,28,29,44].

LIMITATIONS OF THE STUDY: Our study has several limitations worth noting. It is a single center study, limited by a small sample size of 82 sets, use of antibiotics before drawing blood cultures, the number of bacteria depended on the amount of inoculated blood, lack of blood cultures in the control group, and positive cultures in the sepsis group, as well as possible contamination of samples during collection of blood and further laboratory testing.

## 5. Conclusions

FISH demonstrated greater sensitivity in comparison with Gram staining. Both methods enable the detection of bacteria in media culture that did not show growth. Both methods revealed the presence of bacteria in the blood of patients with sepsis and the control group, which confirms the reports to date regarding the constant ‘physiological bacteremia’. Further studies are necessary to distinguish between contamination, asymptomatic bacteremia, and fully symptomatic clinical sepsis.

## Figures and Tables

**Table 1:** Clinical characteristics of studied patient groups.

	Sepsis Group – Blood Cultures		Control Group (*n* = 57)
	Negative (*n* = 82)	Positive (*n* = 8)	
Sex (male/female)	0.64 (± 0.2)	0.85 (± 0.4)	0.31 (± 0.1)
Age (year)	69.4 (± 11.2)	58.8 (± 9.4)	39.6 (± 7.9)
BMI (kg/m^2^)	22.7 (± 4.8)	23.5 (± 3.8)	24.5 (± 1.9)
SOFA score	7.9 (± 4.1)	6.6 (± 4.7)	N/A
WBC (10^9^/L)	17.2 (± 10.9)	18.1 (± 11.3)	4.5 (± 0.8)
CRP (mg/L)	194.1 (± 140.5)	175.5 (± 150.8)	1.4 (± 0.6)
PCT (ng/mL)	5.9 (± 0.6)	5.2 (± 0.7)	N/A

SOFA score - sepsis-related organ failure assessment; WBC – White Blood Cells; PCT – procalcitonin; CRP - C-reactive protein; BMI - Body Mass Index.

**Table 2:** Quantitative comparison of positive and negative results obtained by Gram staining and FISH methods for blood samples, aerobic and anaerobic bottles in the group of patients with sepsis and for blood samples in the control group.

[%]	FISH		
**Gram stain**	**Blood Samples – Control Group**		
		bacteria not found	bacteria visualized
	bacteria not found	35.1	26.3
	bacteria visualized	0.0	38.6
	**Blood Samples – Sepsis Group**		
		bacteria not found	bacteria visualized
	bacteria not found	24.4	13.4
	bacteria visualized	0.0	62.2
	**Aerobic Bottles Samples – Sepsis Group**		
		bacteria not found	bacteria visualized
	bacteria not found	43.9	20.7
	bacteria visualized	0.0	35.4
	**Anaerobic Bottles Samples – Sepsis Group**		
		bacteria not found	bacteria visualized
	bacteria not found	24.4	45.3
	bacteria visualized	11.0	19.3

The differences were statistically significant (*p* < 0.05).
